# Association of *ESR1* Germline Variants with *TP53* Somatic Variants in Breast Tumors in a Genome-wide Study

**DOI:** 10.1158/2767-9764.CRC-24-0026

**Published:** 2024-06-27

**Authors:** Nijole P. Tjader, Abigail J. Beer, Johnny Ramroop, Mei-Chee Tai, Jie Ping, Tanish Gandhi, Cara Dauch, Susan L. Neuhausen, Elad Ziv, Nereida Sotelo, Shreya Ghanekar, Owen Meadows, Monica Paredes, Jessica L. Gillespie, Amber M. Aeilts, Heather Hampel, Wei Zheng, Guochong Jia, Qiang Hu, Lei Wei, Song Liu, Christine B. Ambrosone, Julie R. Palmer, John D. Carpten, Song Yao, Patrick Stevens, Weang-Kee Ho, Jia Wern Pan, Paolo Fadda, Dezheng Huo, Soo-Hwang Teo, Joseph Paul McElroy, Amanda E. Toland

**Affiliations:** 1 Department of Cancer Biology and Genetics, The Ohio State University College of Medicine, Columbus, Ohio.; 2 The City College of New York, City University of New York, New York, New York.; 3 Cancer Research Malaysia, Subang Jaya, Selangor, Malaysia.; 4 Division of Epidemiology, Vanderbilt Epidemiology Center, Vanderbilt-Ingram Cancer Center, Nashville, Tennessee.; 5 Biomedical Sciences, The Ohio State University College of Medicine, Columbus, Ohio.; 6 The Ohio State University Medical School, Columbus, Ohio.; 7 The Ohio State University Wexner Medical Center, Clinical Trials Office, Columbus, Ohio.; 8 Department of Population Sciences, Beckman Research Institute of City of Hope, Duarte, California.; 9 Helen Diller Family Comprehensive Cancer Center, University of California, San Francisco, San Francisco, California.; 10 Department of Medicine, University of California, San Francisco, San Francisco, California.; 11 Institute for Human Genetics, University of California San Francisco, San Francisco, California.; 12 The Ohio State University Comprehensive Cancer Center, Columbus, Ohio.; 13 Department of Internal Medicine, Division of Human Genetics, The Ohio State University, Columbus, Ohio.; 14 Department of Medical Oncology and Therapeutics Research, City of Hope National Medical Center, Duarte, California.; 15 Department of Biostatistics and Bioinformatics, Roswell Park Comprehensive Cancer Center, Buffalo, New York.; 16 Department of Cancer Control and Prevention, Roswell Park Comprehensive Cancer Center, Buffalo, New York.; 17 Slone Epidemiology Center at Boston University, Boston, Massachusetts.; 18 City of Hope Comprehensive Cancer Center, Duarte, California.; 19 Department of Integrative Translational Sciences, City of Hope, Duarte, California.; 20 Bioinformatics Shared Resource, The Ohio State University Comprehensive Cancer Center, Columbus, Ohio.; 21 School of Mathematical Sciences, Faculty of Science and Engineering, University of Nottingham Malaysia, Semenyih, Selangor, Malaysia.; 22 Genomics Shared Resource, The Ohio State University Comprehensive Cancer Center, Columbus, Ohio.; 23 Department of Public Health Sciences, University of Chicago, Chicago, Illinois.; 24 Faculty of Medicine, University Malaya Cancer Research Institute, University of Malaya, Kuala Lumpur, Malaysia.; 25 Department of Biomedical Informatics, The Ohio State University Center for Biostatistics, Columbus, Ohio.

## Abstract

In breast tumors, somatic mutation frequencies in *TP53* and *PIK3CA* vary by tumor subtype and ancestry. Emerging data suggest tumor mutation status is associated with germline variants and genetic ancestry. We aimed to identify germline variants that are associated with somatic *TP53* or *PIK3CA* mutation status in breast tumors. A genome-wide association study was conducted in 2,850 women of European ancestry with breast cancer using *TP53* and *PIK3CA* mutation status (positive or negative) as well as specific functional categories [e.g., *TP53* gain-of-function (GOF) and loss-of-function, *PIK3CA* activating] as phenotypes. Germline variants showing evidence of association were selected for validation analyses and tested in multiple independent datasets. Discovery association analyses found five variants associated with *TP53* mutation status with *P* values <1 × 10^−6^ and 33 variants with *P* values <1 × 10^−5^. Forty-four variants were associated with *PIK3CA* mutation status with *P* values <1 × 10^−5^. In validation analyses, only variants at the *ESR1* locus were associated with *TP53* mutation status after multiple comparisons corrections. Combined analyses in European and Malaysian populations found *ESR1* locus variants rs9383938 and rs9479090 associated with the presence of *TP53* mutations overall (*P* values 2 × 10^−11^ and 4.6 × 10^−10^, respectively). rs9383938 also showed association with *TP53* GOF mutations (*P* value 6.1 × 10^−7^). rs9479090 showed suggestive evidence (*P* value 0.02) for association with *TP53* mutation status in African ancestry populations. No other variants were significantly associated with *TP53* or *PIK3CA* mutation status. Larger studies are needed to confirm these findings and determine if additional variants contribute to ancestry-specific differences in mutation frequency.

**Significance:**

Emerging data show ancestry-specific differences in *TP53* and *PIK3CA* mutation frequency in breast tumors suggesting that germline variants may influence somatic mutational processes. This study identified variants near *ESR1* associated with *TP53* mutation status and identified additional loci with suggestive association which may provide biological insight into observed differences.

## Introduction

*TP53* and *PIK3CA* are among the most frequently mutated genes in breast tumors (1). The frequency of somatic mutations in these genes varies by tumor subtype as well as ancestry (1–4). Pan-cancer and breast cancer–specific studies have found that tumors arising in individuals of African ancestry (AFA), particularly West African ancestry, are more likely to have somatic *TP53* mutations and less likely to have somatic *PIK3CA* mutations than tumors arising in individuals of European ancestry (EUR; refs. 2–9). *TP53* somatic mutations are more common in triple-negative [estrogen receptor (ER) negative, progesterone receptor (PR) negative, HER2 negative] breast cancers (TNBC), while *PIK3CA* mutations are more common in hormone receptor (HR)-positive HER2^−^ tumors. However, even after adjusting for breast cancer subtype, ancestral differences in *TP53* and *PIK3CA* somatic mutation frequencies persist for some subtypes (2–4, 10). For example, one study found that 39% of HR^+^ HER2^−^ tumors from individuals of AFA had *TP53* alterations compared with 24% of those of EUR (*P* value 0.005; ref. 8). Similarly, in HR^+^ HER2^−^ tumors *PIK3CA* somatic mutations are less frequent in individuals of AFA (26%) versus EUR (42%; *P* value 0.001; ref. 8). The biological mechanisms leading to the observed differences in *TP53* and *PIK3CA* somatic mutation frequency across populations and breast tumor subtypes are not understood.

*TP53* encodes transcription factor TP53 and is mutated in a high proportion of breast and other cancers, resulting in altered expression of genes important for response to cellular stress and apoptosis. Unlike many genes involved in tumorigenesis, *TP53* can have either loss-of-function (LOF) mutations, which lead to total loss of the ability of the protein to transactivate, or gain-of-function (GOF) mutations, which result in TP53 binding to new promoters to activate genes not typically associated with TP53 (11, 12). TP53 is a tetramer but can also bind to related proteins TP63 and TP73 (13). Some TP53 tumor-associated mutations act in a dominant-negative manner where the mutant version of the protein interferes with the function of wildtype proteins in the tetramer. In previous studies, we found that in breast tumors with *TP53* mutations, those from AFA women were less likely to have GOF mutations than those from EUR women (14). Mutations without dominant-negative activity were associated with TNBC and ER-negative (ER^−^) status. These data suggest that types of *TP53* mutations in breast tumors differ by self-reported race and tumor subtypes which may be due to different functional consequences of these mutations within cells.

While most somatic events in tumors are likely due to exogenous or endogenous mutators, recent evidence suggests that germline variants may influence the type and burden of somatic changes. Tumor mutational burden, caused in part by somatic mutations in DNA repair genes, is a polygenic trait with an estimated 13% of the variation explained by common germline variants (15). Some tumor mutational signatures are associated with common inherited variants in genes such the apolipoprotein B mRNA editing enzyme catalytic polypeptide (APOBEC) mutation signature and variants in *GNB5* (16). Pathogenic variants (PV) in high-risk cancer susceptibility genes also associate with the presence of somatic mutations and specific mutational signatures. Breast tumors arising in individuals with a germline *BRCA1* PV have more frequent occurrence of somatic *TP53* mutations compared with those without a *BRCA1* PV (17–20). Breast and ovarian tumors arising in individuals with *BRCA1* and *BRCA2* PVs typically show homology directed repair deficiency signatures (20, 21).

On the basis of these studies, we hypothesized that the germline genetic background of an individual can influence specific mutational processes, tumor promotion, and/or mutations in specific cancer-related genes during tumorigenesis, any of which could lead to the observed differences in the frequency of key cancer driver mutations by ancestry (22). The goal of this study was to identify inherited common germline variants (G) that are associated with *TP53* or *PIK3CA* somatic mutation status (M) in breast tumors using a **G**ermline Variant by **M**utation (GxM) genome-wide association study (GWAS) design to assess the influence of genetic background on mutation frequency of these genes.

## Materials and Methods

### Ethics Approval and Consent to Participate

This study was approved by the Ohio State University (OSU) Cancer Institutional Review Board (IRB; protocol number 2005C0082). All data and samples were from deidentified individuals who had undergone informed consent for participation in research studies.

#### Nigerian Study

The City of Hope (COH) IRB and the University of Chicago IRB-approved study for participants enrolled at their respective sites.

#### COH Latina Study

One hundred and twenty Latina patients with breast cancer seen at COH in Duarte, California were included in this study. All participants signed a written informed consent approved by the COH IRB.

#### Malaysian Breast Cancer Study

Malaysian Breast Cancer Study (MyBrCa) was approved by the Independent Ethics Committee, Ramsay Sime Darby Health Care (reference no: 201208.1), and the Medical Ethics Committee, University Malaya Medical Centre (reference no: 842.9).

### Discovery Breast Cancer Datasets

Existing datasets of women with breast cancer from The Cancer Genome Atlas (TCGA; ref. 1), Molecular Taxonomy of Breast Cancer International Consortium (METABRIC; ref. 23), and the Welcome Trust Sanger Institute (24) were used for the discovery GxM GWAS. Each study had existing genome-level single-nucleotide variant (SNV) genotyping data, somatic mutation data for *TP53* and *PIK3CA*, and associated clinical and tumor details such as self-reported race/ethnicity, age at diagnosis, and ER, PR and HER2 status (Supplementary Tables S1–S4).

### *PIK3CA* and *TP53* Somatic Mutation Classification

For discovery and validation analyses, *PIK3CA* mutation status (yes or no) was defined for the following phenotypes: any non-LOF somatic variant in *PIK3CA*, any activating/hotspot mutation (25), or specific activating mutations (e.g., p.E542K, p.E545K, p.H1047R/L). *TP53* mutation status was classified as the presence of any somatic variant in a coding exon or splice-site (yes/no), and variants resulting in *TP53* LOF or GOF as described previously (Supplementary Table S5; Supplementary Data S1; refs. 14, 26). Somatic variants that resulted in a synonymous change and were not predicted to affect splicing were not considered to be a mutation. GOF mutations displayed one or more of the following phenotypes in functional studies: interference with TP63 or TP73 activity, transactivation of genes repressed by wildtype TP53, or cooperation with oncogenes in rat or mouse embryonic fibroblasts. *TP53* LOF variants were those that abolished transactivation activity and/or resulted in altered splicing, frameshift, or nonsense changes. *TP53* somatic missense variants with insufficient data to functionally score as LOF or GOF were called unknown and were not included in LOF or GOF specific analyses (Supplementary Table S5). Larger copy-number loss of *TP53* was not included as a mutation functional category in the analyses due to lack of annotated data for multiple datasets. Controls for each analysis were individuals with breast cancer with no somatic mutation in the gene being assessed.

### Ancestry and GWAS Analyses

PLINK (RRID:SCR_001757) was used to merge datasets, filter, and analyze data. Ancestry SNVs for principal component analyses (PCA) were determined using the Affymetrix annotation accomplished by subtracting the minor allele frequency (MAF) from each of four populations (Han Chinese in Beijing, Yoruba in Ibadan, Northern Europeans from Utah, Japanese in Tokyo) in the annotation in a pairwise manner and taking the top 1,000 SNVs from each comparison. This resulted in the use of 4,486 unique “ancestry” SNVs; 4,212 of those had a MAF of greater than 1%. PCA were performed on these 4,212 SNVs to identify individuals of non-EUR (e.g., those who did not cluster with the EUR group); these individuals were removed from the discovery analyses and were included in the validation studies (Supplementary Fig. S1A–S1C and S2A–S2D). There was a high concordance of ancestry assignment with self-reported race. Filtering also included removal of SNVs with MAF less than 0.01, male participants, and samples and SNVs with greater than 10% missing values. Imputed SNVs were not included. SNVs showing Hardy Weinberg equilibrium *P* values less than 1 × 10^−50^ were also removed. Association analyses were run on a final set of 2,850 females of EUR and 739,537 SNVs with PLINK using a logistic model with a covariate for study. An additive model was assumed. *P* values were FDR corrected and visualized using R (27). QQ Plots for each analysis were generated using R (Supplementary Fig. S3A–S3C and S4A–S4E).

### Selection of Variants for Validation Analyses

**FIGURE 1:**
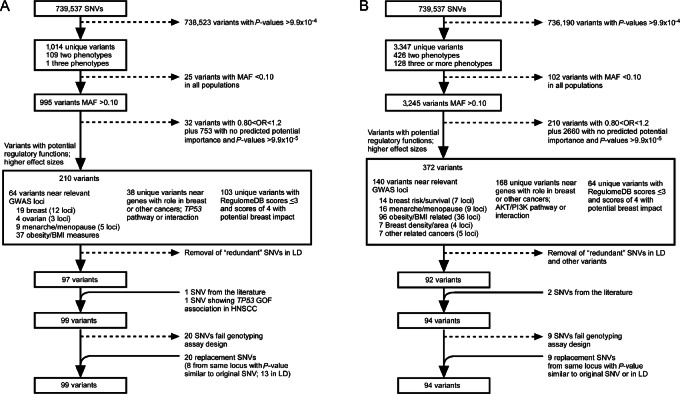
Variant selection for validation studies. Flow chart for variant prioritization for validation studies is shown for *TP53* mutation associations (**A**) and *PIK3CA* mutation associations (**B**). Variants were first filtered by *P* values, MAF, and location near relevant GWAS loci. Variants were then filtered by ORs, location relative to gene with role in breast cancer, TP53 or PI3K/AKT pathway and then by RegulomeDB score. Variants are only counted once but may fall within one or more categories.

Variants were prioritized for validation studies through multiple qualitative and quantitative filtering steps (Supplementary Tables S6 and S7; Fig. 1A and B). Information used to rank variants included *P* values <1 × 10^−4^, ORs, MAF greater than 10% for estimated power detection in at least one of three populations (European, African, or East Asian), allele frequency differences by ancestry, proximity to a variant identified in GWAS for breast cancer risk or other relevant phenotypes (e.g., other cancers, age at menarche, obesity), proximity to a gene showing a role in tumorigenesis, and mapping to a functionally active region (e.g., transcription start site, active chromatin markers, estimated or actual transcription factor binding site, disruption of a transcription factor binding motif, chromatin immunoprecipitation sequencing region for breast cancer cell line, characterized gene enhancer, characterized promoter region, or expression quantitative trait locus). Online resources used for *in silico* screening of candidate SNVs included UCSC Genome Browser (RRID:SCR_005780; ref. 28), GTEx Portal (RRID:SCR_001618; ref. 29), RegulomeDB v.2.0.3 (RRID:SCR_017905; ref. 30), HaploReg v4.0 (RRID:SCR_006796; ref. 31), and dbSNP (RRID:SCR_002338; ref. 32). In addition to variants chosen from the discovery GxM GWAS findings, additional variants were analyzed including two SNVs mapping near *SETD9/MAP3K1* previously shown to be associated with *PIK3CA* somatic mutations in breast cancer (33), an *XPC* variant rs2228001 previously shown to be associated with *TP53* mutation status (34), and a variant in *AURKA* (rs2273535) associated with somatic *TP53* mutations in mouse studies (35). When a genotyping assay for a variant could not be designed for technological reasons, another variant from that locus or a variant in high linkage disequilibrium (LD; *r*^2^ > 0.8) with the original variant was included as a replacement. For validation sample sets with GWAS-level genotyping data, the original variant and the replacement variant were both included in analyses.

### Validation Genotyping

Validation genotyping for 188 SNVs of interest (95 for *TP53* and 93 for *PIK3CA*) was completed for cohorts without existing genome-wide genotyping data including individuals from the Stefanie Spielman Breast Cancer Cohort (*n* = 144), OSU Total Cancer Care (TCC; *n* = 352) and the COH Latina Breast Cancer Study (*n* = 120) using a Fluidigm HD Biomark in a 96 × 96 format in the OSU Comprehensive Cancer Center (CCC) Genomics Shared Resource (GSR; Supplementary Tables S8 and S9). Each genotyping plate contained two duplicate DNA samples, three no-template controls (water), and one control DNA sample genotyped on all plates. DNAs that failed for more than 10% of SNVs from a plate were repeated and if failed again were removed from analysis. SNVs that failed for more than 10% of samples or failed to consistently form three clear genotyping groups were removed from analyses. For genetic ancestry, 96 SNVs were chosen for genotyping from existing ancestry informative marker (AIM) panels (refs. 36–38; Supplementary Tables S10–S12). Of the 96 AIM SNVs, two were removed for poor genotyping performance.

### Somatic Mutational Analyses

For the validation studies, *TP53* and *PIK3CA* mutational status from the clinical testing reports or targeted or exome sequencing of tumor DNA was available for breast cancer cases from the COH Latina Breast Cancer Study, TCGA, and a subset of the TCC cases. For cases in which mutation status was not known, tumor tissue or DNA was available from the Spielman Breast Cancer Cohort and the TCC program for mutational analysis.

### Sanger Sequencing Mutational Analysis

Tumor samples lacking existing somatic mutation data (*n* = 126 for *TP53*, *n* = 184 for *PIK3CA*) were screened for somatic mutations in *TP53* coding exons (exons 2–10) and *PIK3CA* exons 4, 9, and 20 using Sanger sequencing. Tumor DNA (10–20 ng) was PCR amplified and products were confirmed for size by gel electrophoresis (Supplementary Table S13). PCR products were Exo/SAP-IT treated and Sanger sequenced in both forward and reverse directions by the GSR. Sequence chromatograms were evaluated for mutations using DNASTAR Lasergene v.17 (RRID:SCR_000291) by two different laboratory members.

### GxM Validation Analyses

Data used for validation of key findings included genotype and tumor mutational data from individuals of non-EUR from the three discovery datasets as well as samples (germline and/or tumor DNA) or existing data from 1,285 individuals of multiple ancestries from the METABRIC (*n* = 166), Stefanie Spielman Breast Cancer Cohort (*n* = 144), OSU TCC (*n* = 352), a Nigerian breast cancer study (*n* = 100), the COH Latina Breast Cancer Study (*n* = 120), TCGA (*n* = 302), and a TCGA study (“Banerji study”) of women from Mexico and Vietnam (*n* = 101; Supplementary Tables S2–S4 and S14–S18; refs. 3, 4, 39, 40). Genetic ancestry by PCA classified 341 women as AFA (26.5%), 572 women as EUR (44.5%), and 133 women as East Asian ancestry (EAS; 10.4%). The remainder of women (18.6%) were admixed [falling between principal component (PC) clusters], most of whom self-identified as Hispanic/Latino. Because of some missing genotypes, not every variant had data for all 1,285 individuals.

For association analyses, logistic models were employed with an additive effect for the SNV. Study and ancestry were included as covariates in the models. For the study and ancestry-specific analyses, the study analysis omitted the effect of study, and the ancestry analyses omitted the ancestry PC from the models. Because two different panels were used for ancestry determination, individuals of known ancestry (HapMap, TCGA; RRID:SCR_004563 and RRID:SCR_003193 respectively) were used as anchors for each panel. The PC1/PC2 were rotated so that the known ancestry groups overlapped and the distance from the anchor group was calculated as the PC covariables. For individuals with available genome-wide genotyping data, imputation of validation SNVs not present on the GWAS genotyping panels was performed. Imputation was carried out after removing genotypes with no calls or Y chromosome calls. Eagle (RRID:SCR_015991) was used to phase SNV, and imputation was done using Minimac3 (RRID:SCR_009292). The maximum expected error rate across imputed validation SNPs was 0.086. Formats were converted to PLINK format, and variants with greater than two alleles were removed.

### Independent Validation Studies

SNVs of interest were also assessed independently in two cohorts with existing genotyping and mutation data: 859 women with breast cancer from the MyBrCa (41, 42) and 393 AFA women with TNBC from the Breast Cancer in African Americans: Understanding Somatic Mutations and Etiology (B-CAUSE) study (Supplementary Tables S19 and S20; ref. 43). Validation SNVs for the MyBrCa study were excluded if they had a MAF less than 1% in Malaysian individuals and SNVs were excluded from analyses for the MyBrCa and B-CAUSE studies if they mapped to the X chromosome as these data were unavailable. For the MyBrCa study, association tests were conducted using SNPtest adjusted to information for ancestry (four PCs), age of diagnosis, and ER status. B-CAUSE data came from women who self-identified as Black and were diagnosed with TNBC. The African-ancestry Breast Cancer Genetic (AABCG) is a large breast cancer consortium which provided genome-wide genotyping data for the B-CAUSE study. AFA was confirmed by estimating global AFA using ADMIXTURE (ref. 44; Supplementary Tables S20). As the frequency of somatic *TP53* mutations in the B-CAUSE TNBC cases was high, analyses were run for *TP53* GOF-associated germline variants using individuals with LOF *TP53* mutations and those with no mutations as controls; conversely for *TP53* LOF-associated variants, analyses were run using individuals with GOF plus those with no mutations as controls. Logistic regression was employed with a covariable for study and main effect of SNV genotype for *ESR1* variants for combined analyses of Discovery/EUR validation/MyBrCa and AFR validation/B-CAUSE datasets.

### Data Availability

The majority of data generated or analyzed during this study are included in this published article in Supplementary Tables, in TCGA, dbGAP and/or the following data repositories as listed below. TCGA tumor mutation data and SNV genotyping data are available in dbGAP under accession numbers phs001687.v1.p1, phs000178.v11.p8, and phs002387.v1.p1. METABRIC sequencing data of tumors and SNV genotyping data are available on the European Genome-Phenome archive using accession numbers EGAD0001000164, EGAS00000000083, EGAD00010000158, EGAD00010000266, EGAS00001004518, and EGAD00001006399. The Welcome Trust Sanger Institute data are available in the European Genome-Phenome archive using accessing number EGAS00001001178 and EGAD0010000915. Sequencing data and processed genomic data from the Nigerian breast cancer cases are in dbGAP under study accession number phs001687.v1.p1. Tumor/normal whole-exome sequencing (WES) and RNA-sequencing data and accompanying phenotypic and clinical/histologic data for the COH Latina Breast Cancer Study are deposited in dbGAP (dbGaP Study Accession: phs003218; ref. 39). MyBrCa WES and shallow whole genome sequencing (sWGS) files are available on the European Genome-phenome Archive under the study accession number EGAS00001004518. Access to controlled patient data will require the approval of the MyBrCa Tumour Genomics Data Access Committee upon request to genetics@cancerresearch.my. Sequence and genotyping data for the Banerji and colleagues study (40) are available in dbGAP under accession number phs000369.v1.p1. Summary-level statistics genotyping data for the AABCG study are available at GWAS Catalog (accession number: GCST90296719, GCST90296720, GCST90296721, and GCST90296722). B-CAUSE TNBC sequencing data are in the process being deposited into dbGaP with accession number pending.

## Results

To identify germline variants associated with *TP53* or *PIK3CA* somatic mutations in tumors, we identified existing datasets with GWAS-level germline variant information, somatic mutation information for *TP53* and *PIK3CA*, and demographic and clinical information such as age of diagnosis, tumor subtype defined by hormonal (ER and PR) status and HER2 amplification. Three datasets were identified that fit these criteria (Supplementary Tables S2–S4). After filtering for SNVs with MAF less than 1%, individuals with 10% or higher SNV genotypes missing, SNVs out of Hardy–Weinberg equilibrium (*P* value <1 × 10^−50^) and individuals of non-EUR, 2850 females of EUR with breast cancer and 739,537 SNVs were included in the discovery GWAS for variants associated with *TP53* and *PIK3CA* mutation status.

### Discovery GxM for *TP53* and *PIK3CA* Mutation Status

**FIGURE 2:**
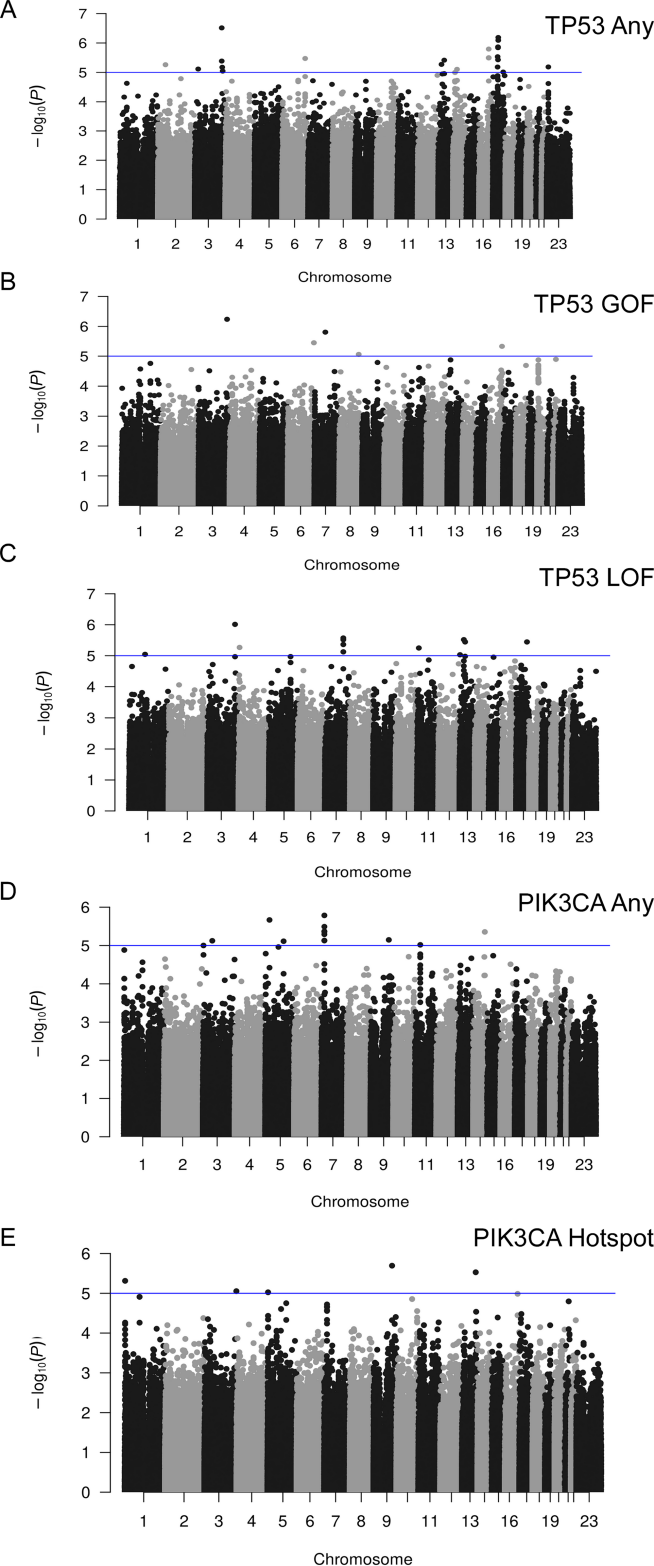
Manhattan plots for *TP53* and *PIK3CA* discovery GxM analyses. Discovery GWAS data for 879 *TP53* mutation carriers and 1,965 breast cancer cases without *TP53* mutations (**A**), 237 cases with *TP53* GOF mutations and 1,965 breast cancer cases without *TP53* mutations (**B**), 536 cases with *TP53* LOF mutations and 1,965 controls (**C**), 1,095 *PIK3CA* mutation carriers and 1,642 breast cancer cases without *PIK3CA* mutations (**D**), and 858 cases with *PIK3CA* activating/hotspot mutations and 1,642 breast cancer cases without *PIK3CA* mutations, are plotted by −log_10_ (*P* values; **E**). Blue lines represent *P* values of less than 1 × 10^−5^. Chromosome numbers are indicated. GXM, germline variant by mutation; GWAS, genome-wide association study; GOF, gain of function; LOF, loss of function.

Analyses for association with any *TP53* mutation, GOF *TP53* mutation, and LOF *TP53* mutation were performed for the 2,850 women of EUR in the discovery dataset in which 30.8% of women had a *TP53* somatic mutation (Table 1; Supplementary Tables S2–S5). Following analysis, no SNV met the genome-wide statistical significance threshold of a *P* value <5 × 10^−8^; four variants were identified with *P* values ≤1.0 × 10^−6^ and 34 variants had *P* values less than ≤1.0 × 10^−5^ across 22 loci (Fig. 2A–C; Supplementary Tables S21 and S22). Two variants showed *P* values of <1.0 × 10^−5^ for more than one *TP53* mutation functional category: rs1561072 for any *TP53* mutation and GOF *TP53* mutations and rs2886631 for any *TP53* mutation and LOF *TP53* mutations.

**TABLE 1:** *TP53* and *PIK3CA* somatic mutation frequencies

Study	Total *N*	TP53 Mutation *N* (%)	TP53 subtype *N* (%)	PIK3CA Mutation *N* (%)	PIK3CA subtype *N* (%)
Discovery	2,850	879 (31%)	GOF 237 (8%)	1,095 (38%)	Activating 858 (30%)
			LOF 536 (19%)		p.E542K 112 (4%)
			Unknown 106 (4%)		p.E545K 193 (7%)
					p.1047R/L 387 (14%)
Validation	1,285	414 (40%)	GOF 110 (11%)	290 (28%)	Activating 235 (23%)
			LOF 277 (27%)		p.E542K 40 (4%)
			Unknown 27 (3%)		p.E545K 58 (6%)
					p.H1047R/L 133 (13%)
MyBrCa	859	369 (43%)	GOF 114 (13%)	247 (29%)	Activating 217 (25%)
			LOF 241 (28%)		p.E542K 20 (2%)
			Unknown 14 (2%)		p.E545K 55 (6%)
					p.H1047R/L 115 (13%)
B-CAUSE	393	365 (93%)	GOF 85 (22%)	9 (2%)	Activating 4 (1%)
			LOF 260 (66%)		p.H1047R/L 2 (0.5%)
			Unknown 20 (5%)		

Abbreviations: *N*, number; %, percent of total number.

Following association analyses for *PIK3CA* mutation status for the 2,850 women in the discovery set, 38% of whom had a *PIK3CA* somatic mutation, no SNV met genome-wide significance of *P* value of <5 × 10^−8^ (Fig. 2D; Table 1). Forty-four SNVs were associated with one or more *PIK3CA* mutation functional category with *P* value <1 × 10^−5^ (Fig. 2E; Supplementary Table S23 and S24). Of these, rs2026801 showed evidence of association (*P* value <1 × 10^−5^) for any *PIK3CA* mutation and activating *PIK3CA* mutations, and rs1712829 showed evidence of association with both p.H1047R and any *PIK3CA* mutation.

### Selection of Variants for Validation Studies

Using *in silico* filtering approaches, all variants with *P* values < 1 × 10^−4^ for any somatic mutation functional category were evaluated for potential inclusion in validation studies. Variants were prioritized for further evaluation by allele frequency in one or more ancestral group (MAF > 10%), potential function using *in silico* prediction models, location near a known GWAS hit for breast cancer or related phenotype (e.g., age of menarche, obesity), location near a gene involved in tumor development, or known relationship to TP53 or PI3K pathways (Fig. 1A and B; Supplementary Tables S6 and S17). Of these, 188 variants from *TP53* (*n* = 95) and *PIK3CA* (*n* = 93) GxM analyses were chosen for validation studies and successfully genotyped in multi-ancestral populations (Supplementary Table S25). For individuals with GWAS-level genotyping data, 119 variants for *TP53* and 106 variants for *PIK3CA* were tested (Supplementary Tables S26–S31).

### Mutation Status and Ancestry in Validation Populations

In the validation datasets, ancestry classifications by PCA yielded 340 AFA individuals, 602 EUR individuals and 134 EAS individuals. The remainder of study individuals (*n* = 209) were considered admixed and not assigned to a specific group; these included individuals of Hispanic/Latino background who demonstrated a high degree of admixture. In the validation datasets, 40% had a *TP53* somatic mutation, and 28% had a *PIK3CA* somatic mutation in their breast tumor (Table 1). The MyBrCa study included 859 women from Malaysia with breast cancer, of whom 43% carried a somatic *TP53* mutation (43%) and 29% had a somatic *PIK3CA* mutation (Table 1). Of the 393 women of AFA with TNBC in the B-CAUSE study, 93% had a *TP53* somatic mutation and only 2.3% had any *PIK3CA* somatic mutations (Table 1).

### Association of Variants at the *ESR1* Locus and *TP53* Mutation Status

Association analyses of validation SNVs were performed separately by ancestry and study. After multiple comparison corrections, variants at the *ESR1* locus were the only ones showing statistically significant evidence of association with *TP53* mutations in at least one validation dataset. In the MyBrCa study, *ESR1* variant rs9383938 showed association with having a *TP53* mutation (OR = 1.81; *P* value 9.8 × 10^−8^) and *TP53* GOF mutation status (*P* value 8.4 × 10^−6^; Table 2; Supplementary Tables S27 and S32). Another *ESR1* locus variant, rs9479090, was also associated with *TP53* mutations in this population (*P* value 2.8 × 10^−7^). Combined analyses of the discovery, EUR validation and MyBrCa studies completed for three variants at the *ESR1* locus, rs9397436, rs9383938, and rs9479090, all showed evidence for association with having one or more *TP53* mutation functional categories after multiple comparisons corrections (Table 2). AFA-specific analyses for these variants showed a trend for association with rs9479090 and having any *TP53* mutation (OR = 1.33, *P* value 0.02; Table 3). None of these variants showed evidence of association in admixed individuals mapping between the European and Asian PCA clusters, most of whom self-identified as Hispanic. Of note, the *TP53*-associated alleles showed lower allele frequency in the EUR and Hispanic populations.

**TABLE 2:** *ESR1* locus variants and *TP53* mutation associations

SNV Ref Allele	rs9397436 T			rs9383938 G		rs9479090 A
*TP53* Mutation	Any	LOF	GOF	Any	GOF	Any
Discovery Cases/Controls	853/1,797	516/1,797	236/1,797	1,073/2,242	292/2,242	1,106/2,303
Discovery OR	1.53	1.48	1.79	1.46	1.71	1.44
Discovery *P*	1.39E-05	6.9E-04	1.1E-04	6.8E-05	3.5E-05	5.4E-05
Discovery MAF	8.4%			10.0%		11.0%
EUR Valid Cases/Controls	217/356	132/356	67/356	130/227	34/227	215/357
EUR Valid OR	1.14	1.04	1.17	1.04	0.96	1.06
EUR Valid *P*	0.55	0.89	0.66	0.87	0.92	0.8
EUR MAF	9.4%			9.4%		10.7%
MyBrCa Cases/Controls	369/490	241/490	114/490	369/490	114/490	369/490
MyBrCa OR	1.43	1.35	1.51	1.81	2.07	1.76
MyBrCa *P*	0.001	0.02	0.01	9.8E-08*	8.4E-06*	2.8E-07*
MyBrCa MAF	37.3%			37.7%		38.1%
Combined Cases/Controls	1,439/2,643	889/2,643	417/2,643	1,572/2,959	440/2,959	1,690/3,150
Combined OR (95% CI)	1.35 (1.17–1.54)	1.24 (1.06–1.46)	1.53 (1.25–1.88)	1.47 (1.31–1.66)	1.59 (1.32–1.90)	1.42 (1.27–1.59)
Combined *P*	1.8E-05^a^	0.007	3.5E-05^a^	2.0E-10^a^	6.07E-07^a^	4.6E-10^a^

Abbreviations: 95% CI, 95% confidence interval; EUR Valid, European ancestry Validation Study; GOF, gain of function; LOF, loss of function; MAF, minor allele frequency; OR, odds ratio; *P*, *P* values; Ref allele, reference allele.

^a^ Significant after multiple comparisons corrections.

**TABLE 3:** *ESR1* variant associations in combined AFA datasets

SNV Ref Allele	rs9397436 T			rs9383938 G		rs9479090 A
TP53 Mutation	Any	GOF	LOF	Any	GOF	Any
Cases/Control	427/333	85/333	296/333	247/146	49/146	401/331
AFA MAF	31%			15%		27%
OR (95% CI)	0.97 (0.69–1.41)	0.59 (0.26–1.17)	1.12 (0.75–1.65)	1.45 (0.94–2.26)	1.42 (0.72–2.75)	1.33 (1.05–1.68)
*P*	0.91	0.16	0.58	0.097	0.3	0.02

Abbreviations: 95% CI, 95% confidence interval; AFA MAF, minor allele frequency in combined African Ancestry datasets; OR, odds ratio, *P*, *P*-value; Ref allele; reference allele.

### Association of Other Loci with *TP53* and *PIK3CA* Mutations

After correcting for multiple comparisons, no other variants were significantly associated with any *TP53* mutation functional category in any of the validation datasets (Supplementary Tables S26–S28, S32, and S33). Variants showing a nonsignificant trend for association in more than one dataset included rs10931697 for *TP53* GOF in the EUR validation, AFR validation, and MyBrCa studies (*P* values 0.008, 0.02, and 0.09, respectively), and rs6709393 (*P* values 0.003 and 0.16) in the MyBrCa and B-CAUSE studies. No SNVs were significantly associated with any *PIK3CA* mutation type in the validation datasets, MyBrCA study or B-CAUSE study (Supplementary Tables S29–S31, S34, and S35).

## Discussion

To our knowledge, this is the first genome-wide breast cancer–specific study to identify germline variants that are associated with *TP53* or *PIK3CA* somatic mutation status. As different types of mutations may have differential effects on cancer-related phenotypes, we also tested for association of specific subcategories of *TP53* (any, LOF, GOF) and *PIK3CA* (any, activating, specific site) mutations with common SNVs. Five variants from the discovery analyses of women of EUR showed suggestive evidence (*P* value <1 × 10^−6^) for association with *TP53* mutation status. Analyses of candidate variants in a Malaysian study, MyBrCa, and combined analyses confirmed that variants at the *ESR1* locus were associated with multiple *TP53* mutation classifications and remained significant after corrections of multiple comparisons.

### *ESR1* Locus Variants, Breast Cancer Risk, and Association with *TP53* Mutation Status

We found evidence that multiple *ESR1* locus variants were associated with *TP53* mutation status. In our discovery study, ten variants at this locus showed a trend toward association (*P* value <1 × 10^−4^) for one or more of the three functional categories of *TP53* mutations. From breast cancer GWAS, multiple variants near *ESR1* have been associated with breast cancer of all subtypes as well as ER^−^ tumors (43, 45–48). Some variants at the *ESR1* locus have been reported to exhibit ancestry-specific association with breast cancer risk (48–50). For example, *ESR1* variant rs140068132 which is thought to have originated in Indigenous Americans, is protective for breast cancer risk (50). In gnomAD, the MAF of *ESR1* variants showing association with having a *TP53* mutation in our study are lowest in individuals of European, Latin American, and South Asian ancestry and are higher in individuals of African and EAS which may explain in part the higher proportion of breast tumors in these populations with *TP53* mutations in these populations.

Variants at the *ESR1* locus were among the first to be associated with breast cancer risk in GWAS (48, 51) and are associated with breast cancer in multiple populations including Chinese, Indian, Nigerian, African American, Malaysian, Latina/Hispanic. European, and Korean (47, 49, 50, 52). These include variants rs9397436 and rs9383938 which were associated with having a tumor with a *TP53* mutation our study (53, 54). Some variants show ancestry-specific differences ORs. Rs2046210, which was originally discovered to be associated with breast cancer in Asian populations, showed a per-allele OR of 1.36 in EAS but ORs close to 1 in EUR and AFA populations (55–57). *ESR1* variants are also associated with specific breast tumor subtypes in GWAS. In EUR-based studies, rs2747652 was associated with HER2-positive/nonluminal breast cancer (58) and rs2757318, rs2046210, and rs9383938 were associated with ER^−^ breast cancer (53, 59). Interestingly, association of rs2046210 with ER^−^ tumors appears to be more pronounced in EUR than EAS (55). Functional mapping of variants across the *ESR1* locus found that multiple variants, including those found in our study, overlap with enhancer regions or show association with *ESR1* expression (45).

In ER^−^ breast tumors, *TP53* and *ESR1* mutations tend to be mutually exclusive (60). This may be due in part to the regulatory relationship between TP53 and ESR1. Mutant *TP53* is correlated with lower *ESR1* gene expression which is thought to be due in part to TP53 binding to the *ESR1* promoter to activate expression (61). Mutant *TP53* tumors have lower estrogen response signatures compared with *TP53* wildtype tumors which may be caused by both decreased transcriptional activation of *ESR1* by mutant TP53 and increased levels of *ESR1*-targeting miRNAs (60). These studies suggest the possibility that mutation of *TP53* may be an early event that promotes lineage toward ER^−^ breast tumors; it is possible that variants at the *ESR1* locus may enhance or reverse this association. Further functional studies are warranted to understand the connection between *ESR1* variants, *TP53* mutational status, and breast cancer subtypes.

### Variants Associated with *TP53* Mutation Status

Other variants in our study showing suggestive evidence of association with *TP53* mutation status included rs17103093 which was associated with any *TP53* mutation phenotype (discovery OR 1.54, *P* value 3.3 × 10^−5^ and combined validation analysis OR 1.4, *P* value 0.03). Rs17103093 maps to an intron of *TACC2*. This variant did not show evidence of association with *TP53* mutations in the MyBrCa study. *TACC2* encodes one of three homologous coiled-coiled proteins; it shows increased expression in higher grade breast tumors and is associated with local recurrence and reduced survival (62, 63). Variants at the *TACC2* locus are associated with risk of low-grade breast cancer, overall breast cancer, and epithelial ovarian cancer (48, 64, 65). Two variants at other loci, rs6703393 and rs6890674, showed consistent direction of association for *TP53* GOF mutations in the discovery analyses (OR 0.79, *P* value 7.5 × 10^−5^) and the MyBrCa study (OR 0.28, *P* value 0.003) but had no evidence of association in the combined validation analyses (*P* values 0.99 and 0.83, respectively). rs6709393 maps near the *RAB17* gene which encodes for a small GTPase associated with invasion (66). rs6890674 is located in the 3′ untranslated region of *CD180*, an orphan Toll-like receptor that is expressed on B cells and is involved in inflammatory and autoimmune diseases (67). Additional studies are needed to determine if these represent real associations.

### Ancestral Differences in *TP53* and *PIK3CA* Mutation Frequencies Across Cancer Types

Associations with genetic ancestry and specific somatic driver mutations have been observed in other cancer types (23, 68). Genetic ancestry is associated with specific somatic driver mutations in *EGFR*, *KRAS,* and *STK11* in lung cancer in individuals of Indigenous American ancestry relative to those of EUR or EAS ancestry (69, 70). *TP53* mutations are found at a higher frequency in individuals of AFA relative to individuals of EUR tumors in multiple tumor types (lung, colon, gastric, human papilloma virus–negative head and neck), suggesting that genetic background and/or differences in exposures/socio-determinants of health may influence selection of *TP53* somatic mutations (71–74). *PIK3CA* somatic mutations also show differences by ancestry in different tumor types. For example, *PIK3CA* mutations have been observed at lower frequencies in bladder tumors arising in EAS individuals and in head and neck squamous cell carcinomas from AFA individuals (74, 75). Conversely, *PIK3CA* mutations are more often observed in colorectal tumors from AFA individuals (76). Variants identified in this study may have utility in explaining *TP53* and *PIK3CA* somatic mutation frequencies arising in different tissues that differ by genetic ancestry. We did not observe any significant AFA-specific associations at the *ESR1* locus after corrections for multiple comparisons, but rs9479090 showed suggestive evidence (*P* value < 0.05).

### Study Limitations

There are limitations to this study. Our discovery analyses were performed in non-Hispanic individuals of EUR, which means that variants enriched in or specific to non-European populations may not have been identified. We were underpowered to determine whether our GxM findings were responsible for the observed differences in breast cancer *TP53* and *PIK3CA* mutation frequency for individuals of non-European populations and for variants associated with specific *PIK3CA* mutations (e.g., p.E542K, p.E545K, and p.H1047R/L). In our validation study, we did not genotype all variants/loci with *P* values of less than 1 × 10^−4^ observed in our discovery set, some of which were not included because of low MAF in one or more populations. As such, we may have missed key variants/loci associated with *TP53* or *PIK3CA* mutation status. The source of somatic mutation information varied widely with some information coming from clinical reports, some from whole genome/WES of tumors, some from targeted sequencing studies, and some from in-house Sanger sequencing studies. Next-generation sequencing is more sensitive than Sanger sequencing for somatic mutations that are present in fewer than 20% of cells or for tumors with a high degree of immune or stromal infiltrate. Our study was based on the premise that *TP53* and *PIK3CA* mutations would be early driver events in tumor development, and mutations in these genes should be present in a high proportion of tumor cells. In a previous study, in which we evaluated types of *TP53* mutation by self-reported race and ethnicity, we found no differences in *TP53* mutation frequency across studies by modality of somatic variation detection suggesting that Sanger sequencing is reasonable for mutation detection of early driver events present in a large proportion of cells (14). Copy number information was not available for a large proportion of tumors; thus, *TP53* mutations due to larger deletions (e.g., chromosome 17p loss) were not included. We expect that a subset of tumors defined as not having a mutation in *TP53* may have had large copy number losses at that locus resulting in the missing of individuals with LOF mutations due to larger deletions.

Across populations, somatic mutations in *TP53* are more common in TNBC and HER2^+^ tumors; conversely, somatic mutations in *PIK3CA* are much more frequent in ER-positive (ER^+^) tumors and luminal breast cancers (2, 4). Even with adjustment based on tumor subtype, it is difficult to sort out the association of the SNV with somatic mutation versus association of the SNV with tumor subtype. Previous studies stratifying by ER^−^ and ER^+^ tumor status have found ancestry differences in mutation frequency for these genes, but this was not the case for all studies stratifying by tumor subtype (4, 8). Future mechanistic studies are needed to determine whether germline variants help drive tumor subtypes that are characterized by certain gene mutations and/or whether germline variants impact a cellular context in which a particular mutation is more likely to be selected and the mutation is important for determining tumor subtype.

## Conclusions

This study provides evidence that *ESR1* germline variants may shape somatic mutation processes or mutation selection of *TP53* in breast tumors. In the future, polygenic risk scores could identify individuals who are at increased risk of mutations in specific genes should they develop breast cancer which may ultimately inform prevention strategies, such as potential vaccination-based prevention for high-risk individuals more likely to carry a specific somatic mutation. Larger multi-ancestry studies are warranted to confirm the study findings and determine whether germline variants explain some of the differences in *TP53* and *PIK3CA* breast cancer mutation frequencies by genetic ancestry. Functional and mechanistic studies are needed to understand the target genes and pathways for variants associated with these mutations in breast tumors.

## Supplementary Material

Supplementary File 1: Supplemental Table References Supplemental Information: References for Supplemental Table S5

Supplemental Tables S1-S35 Supplemental Tables S1-S35

Supplementary Figure 1 Supplementary Figure 1

Supplementary Figure 2 Supplementary Figure 2

Supplementary Figure 3 Supplementary Figure 3

Supplementary Figure 4 Supplementary Figure 4
