# Supplementary material for: Association of ESR1 Germline Variants with TP53 Somatic Variants in Breast Tumors in a Genome-wide Study
Source: Cancer Res Commun. 2024 Jun 27;4(6):1597–608. doi: 10.1158/2767-9764.CRC-24-0026 (PMC11210444; doi:10.1158/2767-9764.CRC-24-0026)
Supplement: Supplementary File 1: Supplemental Table References — Supplemental Information: References for Supplemental Table S5 [file crc-24-0026-s01.docx]

Supplemental Information File 1: References for Supplemental Table S5

1. Giacomelli AO, Yang X, Lintner RE, McFarland JM, Duby M, Kim J, et al. Mutational processes shape the landscape of TP53 mutations in human cancer. Nat Genet. 2018; 50:1381-7.
2. Kotler E, Shani O, Goldfeld G, Lotan-Pompan M, Tarcic O, Gershoni A, et al. A systematic p53 mutation library links differential functional impact to cancer mutation pattern and evolutionary conservation. Mol Cell 2018;71:178-90.
3. Fortuno C, Lee K, Olivier M, Pesaran T, Mai PL, de Andrade KC, et al. Specifications of the ACMG/AMP variant interpretation guidelines for TP53 variants. Hum Mutat. 2021;42;223-36.
4. Fortuno C, Pesaran T, Dolinsky J, Yussuf A, McGoldrick K, Tavtigian SV, et al. An updated quantitative model to classify missense variants in the TP53 gene: a novel multifactorial strategy. Hum Mutat. 2021;42:1351-61.
5. Bouaoun L, Sonkin D, Ardin M, Hollstein M, Byrnes G, Zavadil J, et al. TP53 variations in human cancers: New lessons from the IARC TP53 database and genomics data. Hum Mutat. 2016;37:865-76.
6. Mizuarai S, Yamanaka K, Kotani H. Mutant p53 induces the GEF-H1 oncogene, a guanine nucleotide exchange factor-H1 for RhoA, resulting in accelerated cell proliferation in tumor cells. Cancer Res. 2006;66:6319-26.
7. Slovackova J, Grochova D, Navratilova J, Smarda J, Smardova J. Transactivation by temperature-dependent p53 mutants in yeast and human cells. Cell Cycle. 2010;9:2141-8.
8. Jordan JJ, Inga A, Conway K, Edmiston S, Carey LA, Wu L, Resnick MA. Altered-function p53 missense mutations identified in breast cancers can have subtle effects on transactivation. Mol Cancer Res. 2010;8:701-16.
9. Dittmer D, Pati S, Zambetti G, Chu S, Teresky AK, Moore M, et al. Gain of function mutations in p53. Nat Genet. 1993;4:42-6.
10. Petitjean A, Achatz MI, Borresen-Dale AL, Hainaut P, Olivier M. TP53 mutations in human cancers: functional selection and impact on cancer prognosis and outcomes. Oncogene 2007; 26:2157-65.
11. Kang HJ, Chun S-M, Kim K-R, Sohn I, Sung CO. Clinical relevance of gain-of-function mutations of p53 in high-grade serous ovarian carcinoma. PLoS One. 2013;8:e72609.
12. Monti P, Campomenosi P, Ciribilli Y, Iannone R, Aprile A, Inga A, et al. Characterization of the p53 mutants ability to inhibit p73 beta transactivation using a yeast-based functional assay. Oncogene. 2003;22(34):5252-60.
13. Barta JA, Pauley K, Kossenkov AV, McMahon SB. The lung-enriched p53 mutants V157F and R158L/P regulate a gain of function transcriptome in lung cancer. Carcinogenesis. 2020:41:67-77.
14. Zerdoumi Y, Lanos R, Raad S, Flaman JM, Bougeard G, Frebourg T, Tournier I. Germline TP53 mutations result into a constitutive defect of p53 DNA binding and transcriptional response to DNA damage. Hum Mol Genet. 2017;26: 2591-602.
15. Smith PD, Crossland S, Parker G, Osin P, Brooks L, Waller J et al. Novel p53 mutants selected in BRCA-associated tumours which dissociate transformation suppression from other wild-type p53 functions. Oncogene. 1999;18:2451-9.
16. Xie X, Lozano G, Siddik ZH. Heterozygous p53 (V172F) mutation in cisplatin-resistant human tumor cells promotes MDM4 recruitment and decreases stability and transactivation of p53. Oncogene. 2016;35(36):4798-806.
17. Baroni TE, Wang T, Qian H, Dearth LR, Truong LN, Zeng J et al. A global suppressor motif for p53 cancer mutants. Proc Natl Acad Sci USA. 2004;101:4930-5.
18. Ryan KM, Vousden KH. Characterization of structural p53 mutants which show selective defects in apoptosis but not cell cycle arrest. Mol Cell Biol. 1998;18:3692-8.
19. Scian MJ, Stagliano KE, Anderson MA, Hassan S, Bowman M, Miles MF, et al. Tumor-derived p53 mutants induce NF-kB2 gene expression. Mol Cell Biol. 2005;25:10097-110.
20. Xu J, Reumers J, Couceiro JR, De Smet F, Gallardo R, Rudyak S, et al. Gain of function by mutant p53 by coaggregation with multiple tumor suppressors. Nat Chem Biol. 2011;7:285-95.
21. West AN, Ribeiro RC, Jenkins J, Rodriguez-Galindo C, Figueiredo BC, Kriwacki R, et al. Identification of a novel germ line variant hotspot mutant p53-R175L in pediatric adrenal cortical carcinoma. Cancer Res. 2006;66:5056-62.
22. Kato S, Han SY, Liu W, Otsuka K, Shibata H, Kanamaru R, et al. Understanding the function-structure and function-mutation relationships of p53 tumor suppressor protein by high-resolution missense mutation analysis. Proc Natl Acad Sci USA. 2003;100:8424-9.
23. Göhler T, Jäger S, Warnecke G, Yasuda H, Kim E, Deppert W. Mutant p53 proteins bind DNA in a DNA structure-selective mode. Nucleic Acids Res. 2005;33:1087-100.
24. Friedler A, Veprintsev DB, Hansson LO, Fersht AR. Kinetic instability of p53 core domain mutants: implications for rescue by small molecules. J Biol Chem. 2003;278:24108-12.
25. Wu YH, Tsai Chang JH, Cheng YW, Wu TC, Chen CY, Lee H. Xeroderma pigmentosum group C gene expression is predominantly regulated by promoter hypermethylation and contributes to p53 mutation in lunch cancers. Oncogene 2007;26:4761-73.
26. Tsutsumi-Ishii Y, Tadokoro K, Hanaoka F, Tsuchida N. Response of heat shock element within the human HSP70 promoter to mutated p53 genes. Cell Growth Differ. 1995;6:1-8.
27. Quinn EA, Maciaszek JL, Pinto EM, Phillips AH, Berdy D, Khandwala M, et al. From uncertainty to pathogenicity: clinical and functional interrogation of a rare TP53 in-frame deletion. Cold Spring Harb Mol Case Stud. 2019;5:a003921.
28. Dearth LR, Qian H, Wang T, Baroni TE, Zeng J, Chen SW, et al. Inactive full-length p53 mutants lacking dominant wild-type p53 inhibition highlight loss of heterozygosity as an important aspect of p53 status in human cancers. Carcinogenesis. 2007;28:289–98.
29. Vaughan CA, Frum R, Pearsall I, Singh S, Windle B, Yeudall A, et al. Allele specific gain-of-function activity of p53 mutants in lung cancer cells. Biochem Biophys Res Commun. 2012;429:6-10.
30. Menendez D, Inga A, Resnick MA. The biological impact of the human master regulator p53 can be altered by mutations that change the spectrum and expression of its target genes. Mol Cell Biol. 2006;26:2297–308.
31. Oh SJ, Im MY. The P53 mutation which abrogates trans-activation while maintaining its growth-suppression activity. Mol Cells. 2000;10:386-91.
32. Lang V, Pallara C, Zabala A, Lobato-Gil S, Lopitz-Otsoa F, Farrás R, et al. Tetramerization-defects of p53 result in aberrant ubiquitylation and transcriptional activity. Mol Oncol. 2014; 8:1026-42.
33. Powers J, Pinto EM, Barnoud T, Leung JC, Martynyuk T, Kossenkov AV, et al. A rare TP53 mutation predominant in Ashkenazi Jews confers risk of multiple cancers. Cancer Res. 2020;80:3732-44.
34. Jeffers JR, Pinto EM, Rehg JE, Clay MR, Wang J, Neale G, et al. The common germline Tp53-R337H mutation is hypomorphic and confers incomplete penetrance and late tumor onset in a mouse model. Cancer Res. 2021;81:2442-56.
