# Supplementary material for: Association of ESR1 Germline Variants with TP53 Somatic Variants in Breast Tumors in a Genome-wide Study
Source: Cancer Res Commun. 2024 Jun 27;4(6):1597–608. doi: 10.1158/2767-9764.CRC-24-0026 (PMC11210444; doi:10.1158/2767-9764.CRC-24-0026)
Supplement: Supplementary Figure 1 [file crc-24-0026-s03.docx]

**Supplemental Figures**

**Supplemental Figure 1: Discovery Study Genetic Ancestry Principal Component Analyses**


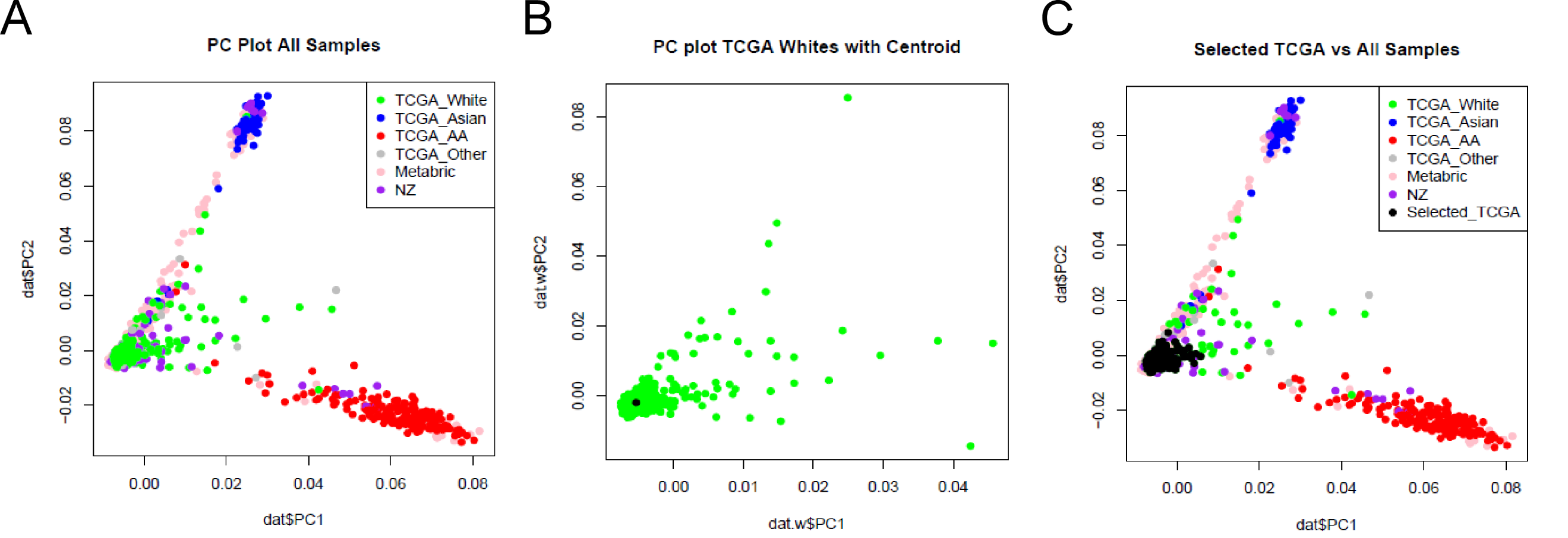


Supplemental Figure 1: Principal component analyses of genetic ancestry are denoted by (A) study and ancestry. (B) PCA of individuals self-identified as white/European race are indicated in green with the centroid indicated as a black dot. (C) Individuals of European genetic ancestry selected for discovery analyses are indicated in black. Individuals in TCGA self-identifying as white/European ancestry TCGA_White (green), black/African ancestry, TCGA_AA (Red), Asian, TCGA_Asian) (Blue) or other race/ethnicity TCGA_Other (Gray). Self-reported race/ethnicity was not included in the METABRIC (pink) or Welcome Trust Sanger (NZ) (purple) studies. PCA, principal component analysis; TCGA, The Cancer Genome Atlas; AA, African Ancestry; METABRIC, Molecular Taxonomy of Breast Cancer International Consortium.
