# Supplementary material for: Association of ESR1 Germline Variants with TP53 Somatic Variants in Breast Tumors in a Genome-wide Study
Source: Cancer Res Commun. 2024 Jun 27;4(6):1597–608. doi: 10.1158/2767-9764.CRC-24-0026 (PMC11210444; doi:10.1158/2767-9764.CRC-24-0026)
Supplement: Supplementary Figure 2 [file crc-24-0026-s04.docx]

**Supplemental Figure 2: Multi-ancestry Validation Study Genetic Ancestry Principal Components Analyses**


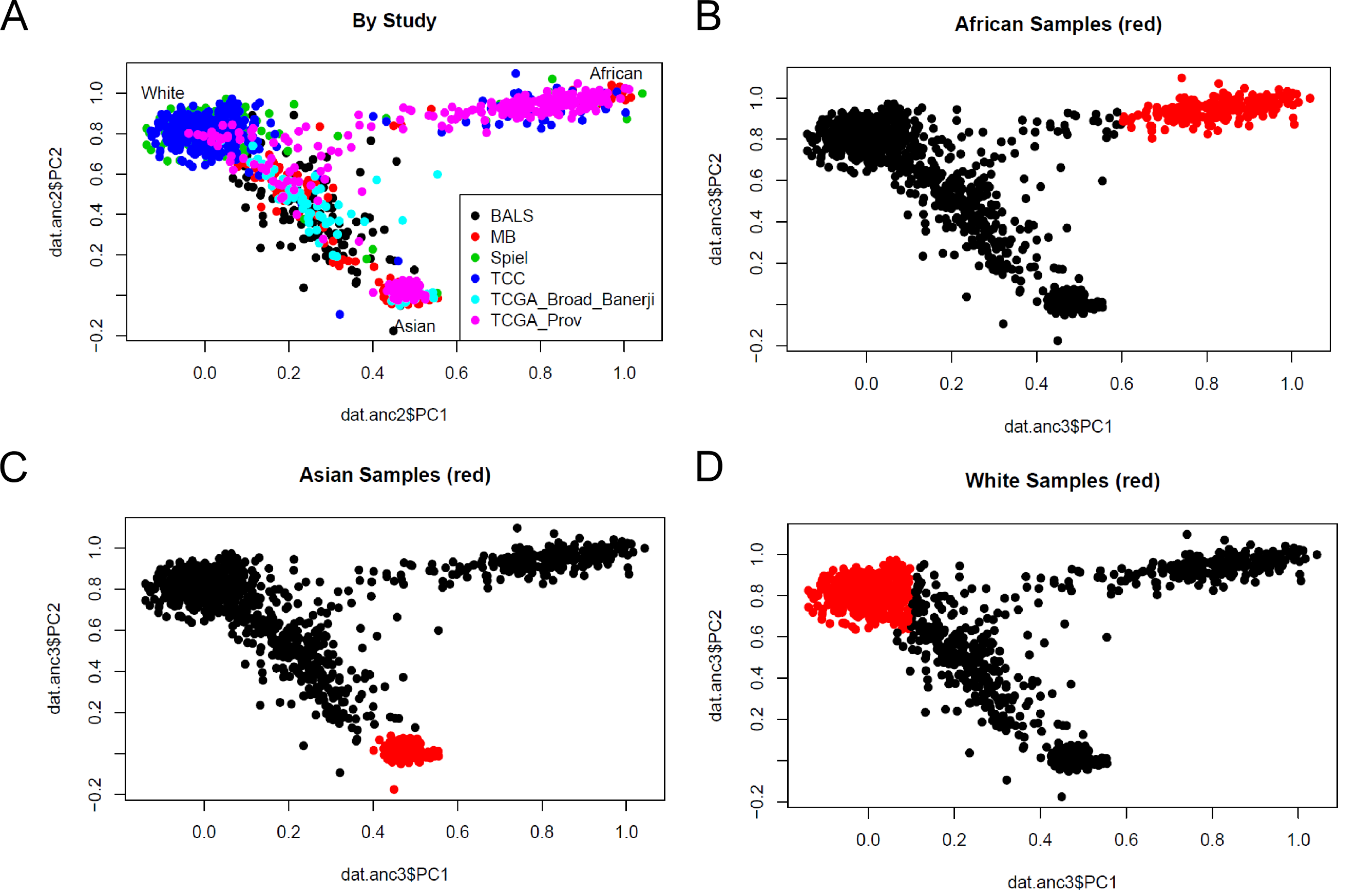


Supplemental Figure 2: Principal component analyses of genetic ancestry are denoted by (A) study cohort or in red by African (B), Asian (C) or European (D) ancestries. Individuals falling between the three major ancestries clusters were assigned as Admixed. BALS, City of Hope Latina Study; MB, METABRIC Molecular Taxonomy of Breast Cancer International Consortium; Spiel, Stefanie Spielman Breast Study; TCC, Total Cancer Care; TCGA_Broad_Banerji, TCGA Banerji study; TCGA_Prov, TCGA Provisional.
