# Supplementary material for: Association of ESR1 Germline Variants with TP53 Somatic Variants in Breast Tumors in a Genome-wide Study
Source: Cancer Res Commun. 2024 Jun 27;4(6):1597–608. doi: 10.1158/2767-9764.CRC-24-0026 (PMC11210444; doi:10.1158/2767-9764.CRC-24-0026)
Supplement: Supplementary Figure 3 [file crc-24-0026-s05.docx]

**Supplemental Figure 3: TP53 QQ Plots**


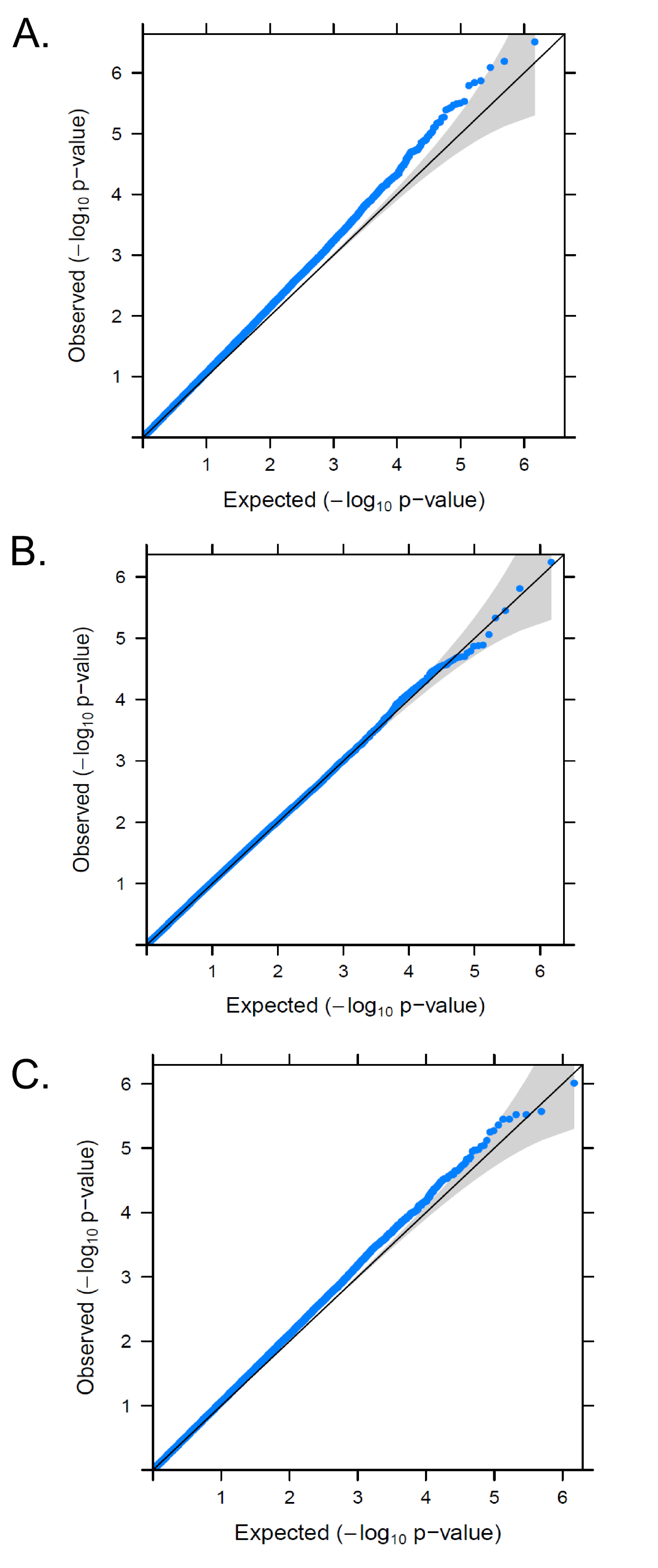


Supplemental Figure 3: *TP53* QQ Plots

QQ plots for the *TP53* GXM are shown for (A) Any *TP53* mutation, (B) GOF *TP53* mutations, and (C) LOF *TP53* mutations. QQ, quantile-quantile; GXM, germline variant by mutation; GOF, gain of function; LOF, loss of function.
