# Supplementary material for: Association of ESR1 Germline Variants with TP53 Somatic Variants in Breast Tumors in a Genome-wide Study
Source: Cancer Res Commun. 2024 Jun 27;4(6):1597–608. doi: 10.1158/2767-9764.CRC-24-0026 (PMC11210444; doi:10.1158/2767-9764.CRC-24-0026)
Supplement: Supplementary Figure 4 [file crc-24-0026-s06.docx]

**Supplemental Figure 4: PIK3CA QQ Plots**


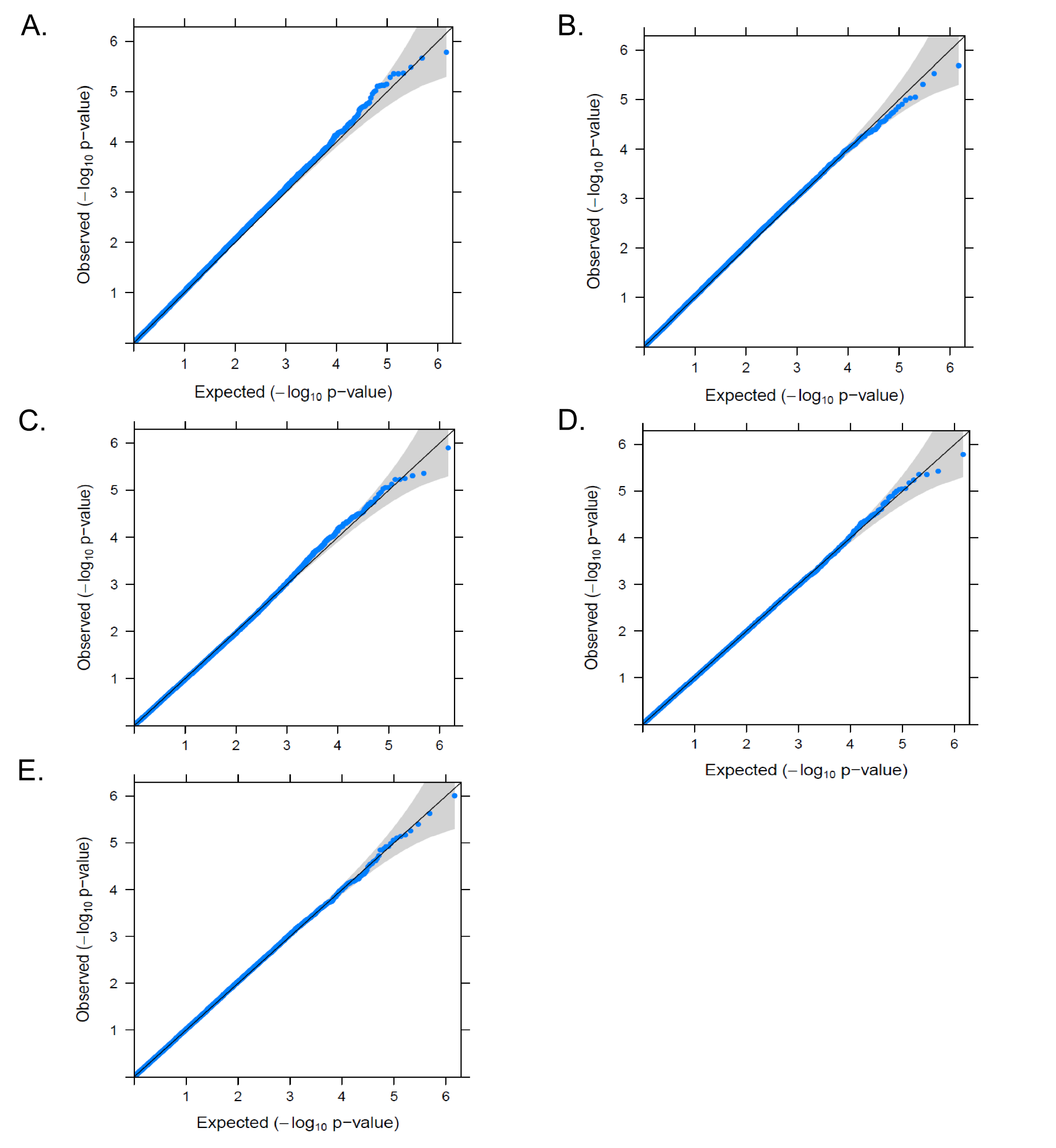


Supplemental Figure 4: *PIK3CA* QQ Plots

QQ plots for the *PIK3CA* GXM are shown for (A) Any *PIK3CA* mutation, (B) Activating/hotspot *PIK3CA* mutations, (C) *PIK3CA* p.E542K mutations, (D) *PIK3CA* p.E545K mutations and (E) *PIK3CA* p.H1047R/L mutations. QQ, quantile-quantile; GXM, germline variant by mutation.
